# Direct glucose production from lignocellulose using *Clostridium thermocellum* cultures supplemented with a thermostable β-glucosidase

**DOI:** 10.1186/1754-6834-6-184

**Published:** 2013-12-21

**Authors:** Panida Prawitwong, Rattiya Waeonukul, Chakrit Tachaapaikoon, Patthra Pason, Khanok Ratanakhanokchai, Lan Deng, Junjarus Sermsathanaswadi, Krisna Septiningrum, Yutaka Mori, Akihiko Kosugi

**Affiliations:** 1 Biological Resources and Post-harvest Division, Japan International Research Center for Agricultural Sciences (JIRCAS), 1-1 Ohwashi, Tsukuba, Ibaraki 305-8686, Japan; 2 Pilot Plant Development and Training Institute (PDTI), King Mongkut’s University of Technology Thonburi (KMUTT), Bangkok, Thailand; 3 School of Bioresources and Technology, King Mongkut’s University of Technology Thonburi (KMUTT), Bangkok, Thailand; 4 University of Tsukuba Graduate School of Life and Environmental Sciences, 1-1-1 Ten-noudai, Tsukuba, Ibaraki 305-8572, Japan

**Keywords:** *Clostridium thermocellum*, β-glucosidases, Glucose production, Biological saccharification, Cellulosomes

## Abstract

**Background:**

Cellulases continue to be one of the major costs associated with the lignocellulose hydrolysis process. *Clostridium thermocellum* is an anaerobic, thermophilic, cellulolytic bacterium that produces cellulosomes capable of efficiently degrading plant cell walls. The end-product cellobiose, however, inhibits degradation. To maximize the cellulolytic ability of *C. thermocellum*, it is important to eliminate this end-product inhibition.

**Results:**

This work describes a system for biological saccharification that leads to glucose production following hydrolysis of lignocellulosic biomass. *C. thermocellum* cultures supplemented with thermostable beta-glucosidases make up this system. This approach does not require any supplementation with cellulases and hemicellulases. When *C. thermocellum* strain S14 was cultured with a *Thermoanaerobacter brockii* beta-glucosidase (CglT with activity 30 U/g cellulose) in medium containing 100 g/L cellulose (617 mM initial glucose equivalents), we observed not only high degradation of cellulose, but also accumulation of 426 mM glucose in the culture broth. In contrast, cultures without CglT, or with less thermostable beta-glucosidases, did not efficiently hydrolyze cellulose and accumulated high levels of glucose. Glucose production required a cellulose load of over 10 g/L. When alkali-pretreated rice straw containing 100 g/L glucan was used as the lignocellulosic biomass, approximately 72% of the glucan was saccharified, and glucose accumulated to 446 mM in the culture broth. The hydrolysate slurry containing glucose was directly fermented to 694 mM ethanol by addition of *Saccharomyces cerevisiae*, giving an 85% theoretical yield without any inhibition.

**Conclusions:**

Our process is the first instance of biological saccharification with exclusive production and accumulation of glucose from lignocellulosic biomass. The key to its success was the use of *C. thermocellum* supplemented with a thermostable beta-glucosidase and cultured under a high cellulose load. We named this approach biological simultaneous enzyme production and saccharification (BSES). BSES may resolve a significant barrier to economical production by providing a platform for production of fermentable sugars with reduced enzyme amounts.

## Background

Many microorganisms capable of producing cellulose and hemicellulose-degrading enzymes have been reported and characterized [1]. *Clostridium thermocellum*, an anaerobic, thermophilic, spore-forming bacterium, is the most potent cellulose-degrading bacterium known to produce cellulosomes [2]. The cellulosomes of *C. thermocellum* contain a surprisingly large variety of enzymes and show attractive enzymatic properties for the degradation of complex plant biomass. In promising attempts to enhance the hydrolytic ability of *C. thermocellum* cellulosomes, designer cellulosomes [2-5] and reconstruction of recombinant cellulosomes [5,6] have been studied using model and natural substrates. On the other hand, *C. thermocellum* produces low levels of cellulosomes (approximately 0.1 g/L) compared to the high level of cellulase secretion (for example, >1 to 10 g protein/L) of *Trichoderma reesei*, which can produce several functionally distinct cellulase components [7]. Although *C. thermocellum* exhibits one of the highest observed microbial growth rates on cellulose [1], it produces less cellulase on a mass basis than aerobic microorganisms. As one promising cost-effective process to resolve these problems, consolidated bioprocessing (CBP) relies on *C. thermocellum* to ferment substrate to desired products (for example, ethanol) in one step without adding externally produced enzymes. This approach is widely recognized as the ultimate configuration for low-cost hydrolysis and fermentation of cellulosic biomass. However, intrinsic features of *C. thermocellum* limit its immediate and direct application to industrial ethanol fermentation from cellulose because it produces only low levels of ethanol and shows weak tolerance to ethanol [8]. In a previous study, we demonstrated remarkable improvements in cellulolytic activity of cellulosomes from the hypercellulolytic *C. thermocellum* S14 strain [9] in combination with a thermostable β-glucosidase from *Thermoanaerobacter brockii* (CglT) [10]. Here, we report on saccharification by *C. thermocellum* cultures supplemented with thermostable β-glucosidases, which we named biological simultaneous enzyme production and saccharification (BSES). BSES required no addition of cellulolytic enzymes. It can directly produce glucose from cellulosic materials due to supplementation of cellulose-degrading cultures with β-glucosidase. Exclusive glucose accumulation of glucose occurred when *C. thermocellum* was cultured with a thermostable β-glucosidase under a high cellulose load. This approach may resolve a significant barrier to economical production of bio-based chemicals and fuels from lignocellulosic biomass.

## Results

### Biological saccharification and glucose production using *C. thermocellum* cultures supplemented with CglT

**Figure 1:**
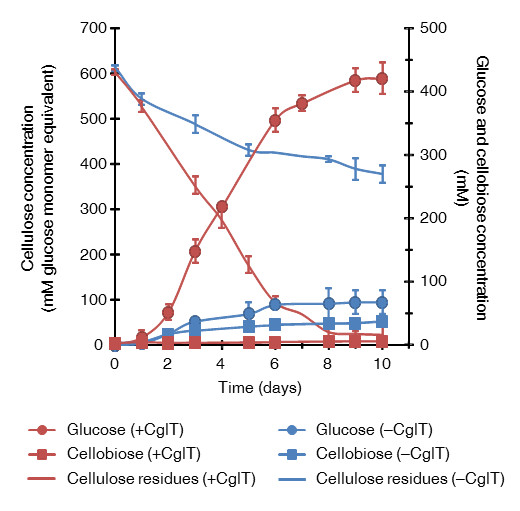
**Biological saccharification using a *C. thermocellum *S14 culture supplemented with CglT.** Monitoring of cellulose hydrolysis and free sugar (glucose and cellobiose) accumulation during cultivation of *C. thermocellum* S14 with or without CglT (+CglT and –CglT, respectively). The units are indicated in mM glucose equivalents. Based on an assumed monomer mass of 162 g/mol of microcrystalline cellulose, a 617 mM glucose equivalent was present initially. Red (+CglT) and blue lines (−CglT) indicate residual cellulose content in culture broth. Error bars represent ± SD (n = 3).

**Figure 2:**
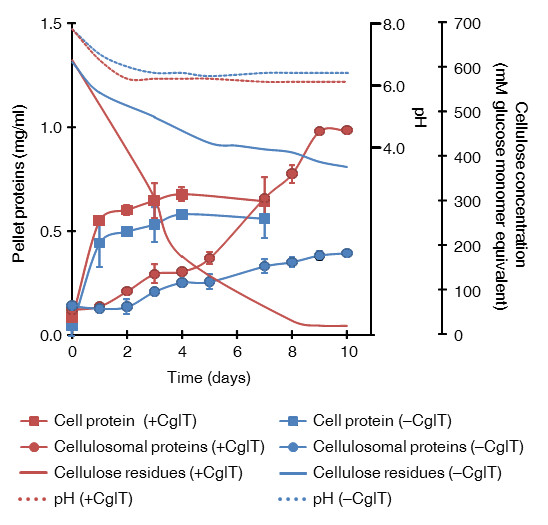
**Profiles of *C. thermocellum *S14 cell growth and released cellulosomal protein in culture broth during biological saccharification.** The protein concentration of the pellet was used as an indication of cell mass and cellulosomal protein during cultivation. *C. thermocellum* was cultured with or without CglT (+CglT and –CglT, respectively) using 100 g/L cellulose (617 mM glucose equivalents). The pH values of each culture are indicated with dotted lines. Red (+CglT) and blue lines (−CglT) indicate residual cellulose content in culture broth. Error bars represent ± SD (n = 3).

CglT of *T. brockii* was characterized as having not only high thermostability, but also relatively high glucose tolerance [11], which suggested it would partner well with *C. thermocellum* cellulosomes. We found that when *C. thermocellum* was cultured with CglT under a high cellulose load, cellulose was effectively converted to glucose, and a high concentration of glucose accumulated in the culture broth. Figure 1 shows the cellulose degradation rate and cellobiose and glucose concentrations in cultures grown with 100 g/L microcrystalline cellulose. In order to allow comparison with glucose production, concentrations are reported in mM glucose equivalents. Based on an assumed monomer mass of 162 g/mol of microcrystalline cellulose, the initial glucose equivalent in the cultures was 617 mM. When *C. thermocellum* was cultured alone on the medium for 10 days, the maximum consumption of cellulose was approximately 234 mM, and most cellulose remained in the broth. The glucose and cellobiose concentrations in the broth were less than 68 mM and 37 mM, respectively, indicating that *C. thermocellum* cultivated on cellulose does not usually produce glucose and cellobiose in the culture broth. In contrast, when *C. thermocellum* S14 was cultured with 30 U/g CglT, it degraded all of the cellulose within 10 days. Furthermore, glucose accumulated to as high as 426 mM in the broth. Based on the cellulose consumption, the majority (70%) was apparently converted to glucose, implying that approximately 30% of the input cellulose was utilized in maintaining cellular homeostasis, such as for energy and enzyme production. The cellobiose content was less than 5.9 mM during cultivation. Cultures both with and without CglT had a pH range of 6.1 to 7.8, with no pH control during cultivation (Figure 2). In this pH range, cellulosomes and CglT appeared to exhibit maximum activities for the substrate.

When cellulose degradation was facilitated by addition of CglT, the amount of free cellulosomal proteins dissociated from the cell surface was higher after 5 days. This phenomenon may be due to release of the cellulosomal proteins by solubilization of cellulose through its effective degradation. In contrast, when *C. thermocellum* S14 was cultured without addition of CglT, although the amount of free cellulosomal protein was slightly higher after 5 days, the majority of cellulosomal protein appeared to associate with the cellulose which still remained at over 370 mM (60 g/L) in the medium (Figure 2). *C. thermocellum* cells reportedly adhere to cellulose through cell surface anchoring proteins [8,12] and biofilms [13]. Cellulosomes are attached to the cell during early log to late exponential growth, and most are found in the medium and attached to cellulose in the stationary phase [8,14]. Thus, glucose production after the stationary phase may be carried out by cooperation between cellulosomes released from the cell surface and the supplemental CglT. Meanwhile, the beginning of glucose accumulation coincided with the stage of cell growth when cells entered stationary phase (after 1 to 2 days of culturing) (Figures 1 and 2). Although the cause of the time lag for glucose accumulation is unclear, cellulosomes attached to the cell may effectively hydrolyze cellulose, allowing the cells to predominantly utilize major hydrolysates such as cellobiose and glucose for proliferation and metabolism during the early log to late exponential growth phase. When the culture reaches the stationary phase after 2 days, cellulosomes dissociated from the cell may continue to hydrolyze cellulose until it is exhausted.

### Optimization of biological saccharification using *C. thermocellum* cultures supplemented with CglT

**Figure 3:**
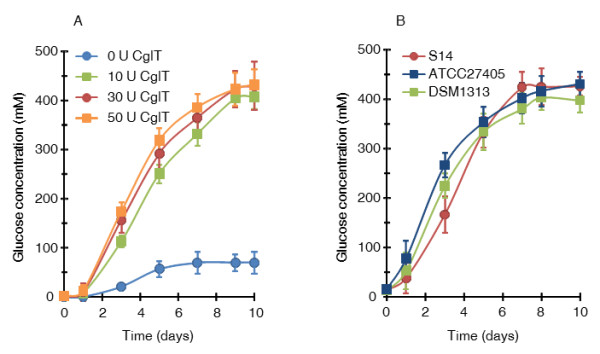
**Optimization of biological saccharification using *C. thermocellum *cultures. (A)** Effect of CglT activity on biological saccharification. *C. thermocellum* S14 cultures were supplemented with 10, 30, or 50 U of CglT per g cellulose or left unsupplemented (0 U) at an initial concentration of 100 g/L cellulose. **(B)** Influence of strain on glucose concentration. *C. thermocellum* cultures were supplemented with 30 U of CglT per g cellulose at an initial concentration of 100 g/L cellulose. *C. thermocellum* ATCC 27405 and DSM 1313 were used as type strains for comparison to the originally isolated strain S14. Error bars represent ± SD (n = 3).

For biological saccharification and glucose production, *C. thermocellum* cultures require supplementation with CglT. Alternatively, BglA (*C. thermocellum*) [15] and Bgl (*Thermoanaerobacterium thermosaccharolyticum*) [10,16], which belong to glycoside hydrolase family-1 (Carbohydrate-Active enZYmes Database; http://www.cazy.org/Glycoside-Hydrolases.html), have been reported as other promising β-glucosidases to enhance cellulose saccharification in combination with cellulosomes. In particular, Bgl was characterized as possessing higher glucose tolerance, which may be an important property for compatibility with *C. thermocellum* cellulosomes under a high cellulose load [16]. Although there were no notable differences in β-glucosidase activity, optimum pH, or glucose tolerance among these three β-glucosidases, the thermostability of CglT was significantly higher at 60°C (Table 1). To assess whether thermostability of β-glucosidases affects biological saccharification and glucose production, *C. thermocellum* cultures grown with 100 g/L cellulose were supplemented with recombinant BglA or Bgl instead of CglT. Supplementation with BglA or Bgl led to lower cellulose degradation ability and glucose production than with CglT, and a higher accumulation of cellobiose was observed for both (Table 1). Thus, high thermostability of the β-glucosidase partnered with *C. thermocellum* was essential for efficient cellulose degradation and high glucose production. To confirm that CglT activity influences glucose production, *C. thermocellum* S14 cultures were supplemented with various amounts of CglT. When 10, 30, and 50 U of CglT per g cellulose were added to the culture, the released glucose concentrations were 408, 429, and 432 mM, respectively (Figure 3A). These results suggest that maximum glucose production was achieved by supplementation of *C. thermocellum* cultures with 30 to 50 U of CglT per g cellulose.

**Table 1:** **Biological saccharification using glycoside hydrolase family-1 β-glucosidases from *C. thermocellum *** **and *T. thermosaccharolyticum***

	**Specific activity (U/mg)**	**Thermal stability (%)**^**a**^	**Optimum temperature (°C)**^**b**^	**Optimum pH**^**b**^	**Glucose inhibition (mM)**^**c**^	**Biological saccharification**	
						**Cellobiose concentration (mM)**^**d**^	**Glucose concentration (mM)**^**d**^
CglT (*T. brockii*)	26.0 ± 0.05	97	60 to 75	6.0 to 7.0	450 ± 10.2	5.9 ± 0.7	425.9 ± 24.7
BglA (*C. thermocellum* ATCC)	19.4 ± 0.05	10	50 to 60	6.0 to 7.0	400 ± 5.1	22.1 ± 1.3	240.7 ± 18.5
Bgl (*T. thermosaccharolyticum* NOI-1)	28.8 ± 0.05	10	60 to 70	6.0 to 7.0	650 ± 3.2	36.4 ± 0.7	304.3 ± 18.5

^a^Thermal stability is indicated as the percentage of remaining β-glucosidase activity after incubation for 24 hours at 60°C; ^b^range reported represents where >90% of the maximal activity was maintained; ^c^glucose inhibition was calculated as the glucose concentration required to inhibit 50% of initial β-glucosidase activity; ^d^biological saccharification was carried out by culturing *C. thermocellum* with each β-glucosidase using 100 g/L crystalline cellulose as described in the Methods section. To allow comparison with glucose production, cellobiose concentration was indicated in mM glucose equivalents. Values are the means of triplicate experiments ± SD.

This study primarily used the originally isolated *C. thermocellum* S14 strain [9]. In order to confirm whether typical type strains of *C. thermocellum* such as ATCC 27405 and DSM 1313 also show high cellulose degradation and glucose accumulation, *C. thermocellum* ATCC 27405 and DSM 1313 supplemented with CglT were cultured with 100 g/L cellulose (Figure 3B). Both *C. thermocellum* strains could completely degrade the cellulose in medium and accumulated approximately 400 mM glucose into the broth, indicating that biological saccharification can be applied to other *C. thermocellum* strains. Nevertheless, there were no big differences in final glucose productivity and cellulose degradation ability between these strains, though glucose accumulation during cultivation of *C. thermocellum* ATCC 27405 and DSM 1313 was slightly faster than that of the S14 strain (Figure 3B). Although it is unclear why moderately late glucose production is observed in *C. thermocellum* S14, it might be related to differences in growth properties on glucose between the S14 strain and the type strains [9]. *C. thermocellum* S14 can grow on glucose medium without a long lag period, suggesting that physiological responses of the S14 strain such as uptake and metabolism of glucose may differ from those of type strains.

**Figure 4:**
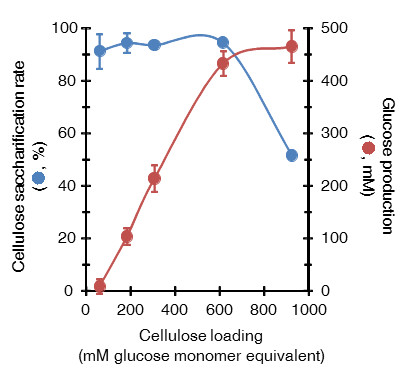
**Relationship between cellulose saccharification ability and glucose production under various cellulose loads.** Biological saccharification was carried out in BM7CO medium containing various concentrations of cellulose: 10 g/L (62 mM glucose equivalent), 30 g/L (185 mM glucose equivalent), 50 g/L (309 mM glucose equivalent), 100 g/L (617 mM glucose equivalent), or 150 g/L (926 mM glucose equivalent). The cellulose saccharification rate was calculated based on the amount of glucose released relative to the dry weight of the input cellulose, respectively. Error bars represent ± SD (n = 3).

High cellulose loading is preferred for industrial processes due to the benefits of lower capital cost and higher titers of desirable products such as ethanol. Thus, high cellulose input should provide enough fermentable sugars for ethanol production. To confirm suitable cellulose loading for biological saccharification, we investigated the relationship between saccharification ability and glucose accumulation. *C. thermocellum* S14 supplemented with CglT was cultured using cellulose inputs of 10, 30, 50, 100, or 150 g/L (Figure 4). High saccharification of 86.5% to 94.6% was obtained in cultures using 10 to 100 g/L cellulose, whereas hydrolysis decreased drastically to 51.6% when substrate input was elevated to 150 g/L. The accumulation of 465 mM glucose and 47 mM cellobiose as hydrolysis products caused more severe end-product inhibition of cellulosomes and β-glucosidases. Cellobiose is the main product of cellulose hydrolysis by *C. thermocellum*. Johnson *et al.* reported complete inhibition of cellulase activity at cellobiose concentration of 20 g/L when microcrystalline cellulose was used as the substrate [17]. *C. thermocellum* cellulosomes were much less sensitive to glucose, as it required 60 g/L of glucose for 35% cellulase inhibition. To a lesser extent, glucose also inhibits *C. thermocellum* cellulosomes [8]. At high glucose concentration (465 mM), the β-glucosidase activity of CglT was inhibited by 50% (Table 1). Fermentation products such as ethanol and acetate that accumulate during cultivation also have negative effects on hydrolysis by *C. thermocellum*[18]. In the present study ethanol concentration was <0.3 g/L in all cases, so ethanol is unlikely to be the cause of inhibition [19]. However, the acetate and lactate as fermentation products cause a pH drop in culture broth; the final pH values of all cultures in the present study were in the range of pH 6.1 to 6.4 in the absence of pH control. These results suggested that the decreased saccharification rate in 150 g/L cellulose might result from a performance decline of cellulosome and CglT due to high accumulation of glucose and cellobiose. On the other hand, high glucose production was obtained in cultures with a cellulose input of 30 to 150 g/L but only 8.3 mM from 10 g/L (Figure 4). These results suggest that *C. thermocellum* may utilize carbon catabolism predominantly to maintain cell homeostasis, growth, and enzyme and energy production when the cellulose concentration in the culture medium is low. Thus, in order to achieve high glucose production and accumulation by biological saccharification, a cellulose load over 10 g/L is also required in the culture medium.

### Biological saccharification and ethanol fermentation using alkali-treated rice straw

To test whether biological saccharification will work for lignocellulosic biomass sources other than pure cellulose, the process was carried out using alkali-pretreated rice straw. The chemical composition of the rice straw obtained in the filtration cake (solid remaining, 47.5% w/w dry weight) after 10% (w/v) NaOH pretreatment at 70°C for 5 days was 85.5 ± 0.4% glucan, 5.2 ± 0.5% xylan, 0.7 ± 0.03% arabinan, and 0.3 ± 0.09% galactan (w/w). The Klason lignin content was 6.9 ± 0.3% (w/w), indicating a delignification ratio of 62.3% of the original lignin content (18.3% w/w).

**Figure 5:**
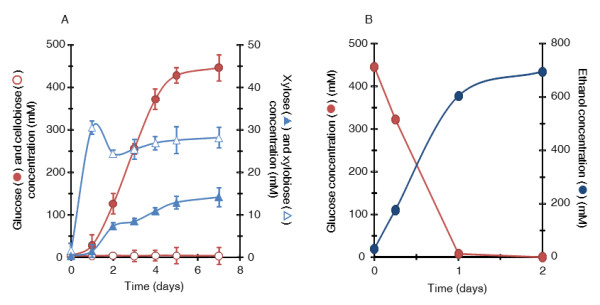
**Biological saccharification and ethanol fermentation profiles using alkali-pretreated rice straw. (A)** Saccharification and **(B)** fermentation. The units are given in mM glucose and xylose equivalents. Initially, based on an assumed monomer mass of 162 g/mol of glucan and 132 g/mol of xylan, 617 mM glucose and 46 mM xylan equivalents were present. Error bars represent ± SD (n = 3).

*C. thermocellum* S14 supplemented with CglT was cultured under a high solid load of 120 g/L alkali-pretreated rice straw (3% moisture content) containing 100 g glucan and 6.1 g xylan. Although the biomass slurry at the beginning had a high viscosity, after supplementation with CglT, viscosity was dramatically reduced. Cellobiose was maintained at a very low concentration, less than 3.9 mM throughout cultivation. Glucose accumulated to 446 mM, corresponding to approximately 72% saccharification of input glucan, which was comparable to tests using pure cellulose (Figure 5A). Xylan in alkali-pretreated rice straw could be degraded to xylobiose and xylose, which accumulated to 28 mM and 14 mM, respectively, as xylose equivalents. Xylan showed a high degree of hydrolysis. In profiles of sugar production, xylan degradation occurred more quickly than glucan hydrolysis, and xylobiose was released as the main product into the broth (Figure 5A). A xylan structural barrier is one of the major mechanisms that limits the accessibility of cellulases to the cellulose; cellulose accessibility is necessary for synergistic cooperation between xylanases and cellulases [20]. The released xylobiose and xylose remained in the culture supernatant because *C. thermocellum* is unable to utilize pentose sugars [8].

In order to evaluate whether the hydrolysis slurry derived from biological saccharification could be immediately used for ethanol fermentation, *Saccharomyces cerevisiae* was directly inoculated to the slurry. No other nutrients were added. The slurry (pH 5.9) contained 446 mM of glucose, *C. thermocellum* cells, and CglT, as well as fermentation products such as lactic acid (2.15 ± 0.02 mg/mL), acetic acid (4.18 ± 0.07 mg/mL), and ethanol (0.13 ± 0.02 mg/mL). Fermentation profiles of ethanol production and glucose consumption were obtained from the slurry (Figure 5B), as well as profiles of cultures grown on the reference fermentation test using medium containing the same concentration of glucose. Ethanol productivity was 695 ± 4 mM, which corresponds to approximately 85% of the theoretical yield, indicating that the slurry obtained from *C. thermocellum* cultures supplemented with CglT can be fermented directly without any inhibition.

## Discussion

Here we describe the first example of biological saccharification with glucose production from lignocellulosic biomass. To achieve this, we used *C. thermocellum* cultures supplemented with a thermostable β-glucosidase. Biological saccharification using *C. thermocellum* requires no addition of cellulase or hemicellulase; however, supplementation with a thermostable β-glucosidase to the culture is essential for glucose accumulation. We named this saccharification process BSES. Although BSES is similar to CBP in whole-cell catalyzing systems using *C. thermocellum*, it focuses on the objective of hydrolysis of lignocellulosic biomass and glucose production. Thus, BSES can be imagined as combining the two processing steps of cellulase production and cellulose hydrolysis, which are ordinarily performed as separate hydrolysis and fermentation (SHF) reactions into a single step [1].

It is known that a combination of cellulosomes and a thermostable β-glucosidase leads to efficient saccharification due to elimination of end-product inhibition under high cellulose loading [10,15,21]. At the same time, the *C. thermocellum* cellulase complex is substantially more effective during microbial hydrolysis than purified cellulase preparations from this microorganism [22]. This phenomenon, called enzyme-microbe synergy, requires the presence of metabolically active cellulolytic microbes, suggesting that the process may require not only the removal of hydrolysis products from the fermentation broth, but also surface phenomena involving adherent cellulolytic microorganisms [22]. Thus, the efficient saccharification and glucose accumulation through BSES may be caused by enzyme-microbe synergy and efficient elimination of end-product inhibition by cellobiose in the fermentation broth with CglT.

*C. thermocellum* has an uptake system capable of assimilating cellodextrins, which are released through simultaneous multiple hydrolysis steps mediated by adjacent cellulosomal subunits [14]. Glucose uptake occurs only in cells grown on the monomer and has been characterized as a low-affinity system compared to the one involved in cellobiose uptake [23]. Growth of *C. thermocellum* ATCC 27405 on glucose was obtained only after an exceptionally long lag of over 100 hours [24], and the cell yield was higher on cellobiose than on glucose [25]. The physiological responses and adaptation related to the long lag period of *C. thermocellum* for utilization of monosaccharides such as glucose are understood as the lack of any beneficial availability of ATP derived from phosphorolytic cleavage by intracellular cellobiose phosphorylase (CbP) and cellodextrin phosphorylase (CdP) during growth on glucose [1,26], and the requirement for induction or deregulation of the utilization pathways involving glucose uptake [1,8]. Thus, the phenomenon of high glucose accumulation appears to be due not only to effective cellulose degradation by cooperation of cellulosomes and CglT, but also to a low-affinity system for glucose uptake by *C. thermocellum*. The phenomenon of high glucose accumulation may also be found in BSES using mesophilic cellulolytic anaerobes possessing similar uptake and phosphorylation (CbP and CdP) systems that are known to be widely distributed in cellulose-utilizing anaerobes, such as *C. cellulovorans*[5,27], *C. cellulolyticum*[28], and *Ruminococcus flavefaciens*[29]. On the other hand, *C. thermocellum* may be better suited for BSES than the mesophilic anaerobes for the following reasons: (1) *C. thermocellum* exhibits substantially higher enzyme production levels and growth rates on crystalline cellulose than any mesophiles [1,30]; (2) thermophiles, including *C. thermocellum*, are considered to be robust microorganisms and the cellulolytic enzymes are stable in thermophilic conditions [31]; (3) the problem of contamination in the presence of high glucose concentrations can be avoided in the growth condition for *C. thermocellum*[8]; and (4) *C. thermocellum* can degrade hemicellulose as well as mesophiles [32-34], but does not utilize pentose sugars, such as xylose and xylobiose.

**Figure 6:**
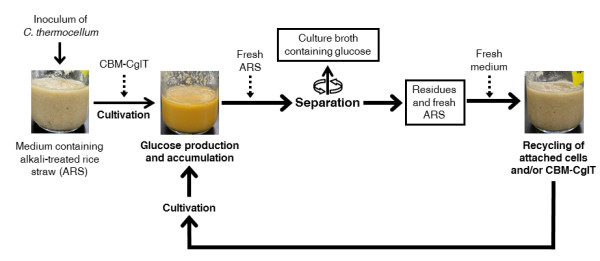
**Schematic of consecutive biological saccharification method based on recycling the hydrolyzed residue.***C. thermocellum* supplementation with CBM-CglT was only carried out in the first biological saccharification round, without further culturing or addition of any enzymes. To recover free cellulosomes, CBM-CglT, and *C. thermocellum* cells, fresh pretreated cellulose substrates were added to the hydrolysis slurry and then reabsorbed from the supernatant. A second round of biological saccharification was performed using enzymes recovered by allowing them to bind to fresh substrate and the hydrolysis residue containing *C. thermocellum* cells. Consecutive biological saccharification using these recycling procedures may be repeated several times. When recombinant *C. thermocellum* strains overexpressing CglT or CBM-CglT are used in consecutive biological saccharification, addition of thermostable β-glucosidases may not be required. The left and center images are the relative initial biomass containing 120 g/L alkaline-pretreated rice straw and the culturing slurry. The right image shows the appearance of attached cells and CBM-CglT during recycling. ARS, alkaline-pretreated rice straw.

One method that may reduce the amount of cellulase used and increase enzymatic productivity is recycling of the cellulases. Many studies have already been carried out on recycling strategies using *T. reesei* cellulases [35]. Cellulase recycling approaches have included recovery of free enzymes from the liquid fraction [36] and introduction of fresh substrate to the liquor fraction, allowing the enzymes to be absorbed to the substrate before separation and hydrolysis [37]. Recently, we developed a recycling method utilizing a combination of *C. thermocellum* cellulosomes and chimeric CBM-CglT created by fusing the carbohydrate-binding module CBM3 from the scaffolding protein CipA into the N-terminal region of CglT [38]. It seems that the concept of recycling an enzyme may easily apply to biological saccharification using *C. thermocellum* and CglT. If CBM-CglT instead of CglT is used during biological saccharification, hydrolysis and glucose production would be carried out for several days, after which cellulosomes, CBM-CglT, and insoluble solid residues bound to *C. thermocellum* cells may be recovered by adding fresh pretreated rice straw to the broth. *C. thermocellum* and CBM-CglT would only be expected to be added during the first round, making additional β-glucosidase or fresh inoculum unnecessary (Figure 6). CglT and CBM-CglT appeared to be suitable enzymes, since both recombinant proteins can be highly expressed as soluble proteins in the periplasmic space of *Escherichia coli* (>14,500 U/L medium) and simply prepared by direct heat treatment (65°C) and cell lysis [39,40]. Alternatively, a *C. thermocellum* expressible BglA was created in shuttle vector pIBglA in an attempt to increase cellulase activity [41]. When these ideas can be introduced to a system for biological saccharification of lignocellulosic biomass, the most cost-effective saccharification system not requiring any enzymes can be established.

## Conclusions

Economical production of bio-based chemicals and fuels from lignocellulosic biomass by enzymatic means continues to require considerable research in terms of both technical and economic aspects. In particular, cellulolytic enzymes continue to be one of the major problems associated with the hydrolysis process. *C. thermocellum* is the most potent cellulose-degrading bacterium known to produce cellulosomes. Biological saccharification using *C. thermocellum* cultures supplemented with thermostable β-glucosidases provide a platform for production of fermentable sugars with drastically reduced requirements for enzyme amounts. This advance is expected to immediately benefit for biomass refinery. To take full advantage of these prospects, future studies will address improvement of *C. thermocellum*, the cellulase system, and thermostable β-glucosidases. The goals are much higher tolerance to glucose, cellobiose, and fermentation products such as ethanol and acetate.

## Methods

### Organisms, media, and growth conditions

The hypercellulolytic strain *C. thermocellum* S14 has been deposited with the National Institute of Technology and Evaluation Patent Microorganisms Depositary (NPMD; Chiba, Japan) as NITE P-627. *C. thermocellum* ATCC 27405 and *T. brockii* ATCC 33075 were obtained from the American Type Culture Collection (ATCC; Manassas, VA, USA). *C. thermocellum* DSM 1313 was obtained from Leibniz Institute DSMZ - German Collection of Microorganisms and Cell Cultures (Braunschweig, Germany). *T. thermosaccharolyticum* NOI-1 was originally isolated from soil and stored in our laboratory [42]. *C. thermocellum* was grown on BM7CO medium [9,43] supplemented with 10 g/L microcrystalline cellulose powder (Sigmacell type 20; Sigma-Aldrich, St Louis, MO, USA) or 10 g/L cellobiose (Sigma-Aldrich). *T. brockii* was grown in modified DSMZ 122 medium supplemented with 5 g/L cellobiose as the carbon source [10]. All BM7CO media were degassed in boiling water and bubbled with high purity carbon dioxide gas. *E. coli* DH5α (Takara Bio, Shiga, Japan), *E. coli* BL21(DE3), and plasmid pET19b (Merck KGaA, Darmstadt, Germany) served as the cloning host, expression host, and vector, respectively. *E. coli* cells were grown at 37°C in Luria-Bertani medium containing ampicillin (50 μg/mL). The sake-brewing yeast *S. cerevisiae* Kyokai no. 3 (K3) was obtained from the National Research Institute of Brewing (NRIB; Hiroshima, Japan). In preculture, the yeast was grown aerobically in static culture at 30°C on complete medium (YPD) containing 20 g/L peptone, 10 g/L yeast extract (Difco Laboratories, Detroit, MI, USA), and 20 g/L glucose.

### Preparation of recombinant β-glucosidases

Preparation of chromosomal and plasmid DNA, and transformation were carried out by standard procedures or according to supplier protocols (Qiagen, Frederick, MD, USA). Constructed plasmid pET19CglT was used to prepare recombinant CglT [10]. We designed oligonucleotide primers to clone *bgl*A from *C. thermocellum* and *bgl* from *T. thermosaccharolyticum* NOI-1. Primer pairs containing artificial restriction enzyme recognition sites (*italics*) were used to amplify fragments of *bgl*A (5′-ATG*CTCGAG*ATGTTTCCTCTAGGTTATAAT-3′ for sense primer with *Xho*I sites, and 5′-ATT*GCTCAGC*TTAAAAACCGTTGTTTTTGA-3′ for antisense primer with *Bpu*1102 sites) from *C. thermocellum* ATCC 27405 and *bgl* (5′-GGAATTC*CATATG* TCGGACTTTAACAAGGA-3′ for sense primer with *Nde*I sites, and AGTCA*CTCGAG*AATGGTCCTAGTGGAAAT-3′ for antisense primer with *Xho*I) from *T. thermosaccharolyticum* NOI-1 by PCR using genomic DNA as template. PCR was performed with PrimeSTAR HS DNA polymerase (Takara Bio) under standard conditions according to the manufacturer’s instructions. The amplified fragments for *bgl*A and *bgl* were respectively inserted between the *Xho*I and *Bpu*1102 sites and the *Nde*I and *Xho*I sites of pET19b to generate pET19BglA and pET19Bgl. Expressed CglT, BglA, and Bgl were purified by nickel affinity column chromatography using Ni-NTA agarose resins and desalted using a desalting column.

### Enzyme and protein assays

Assays for crude and recombinant β-glucosidase were performed at 60°C in 0.1 M sodium acetate buffer (pH 6.0) with 5 mM CaCl_2_ under static conditions for 10 minutes [10]. Determination of β-glucosidase activity was based on measurement of the release of *p*-nitrophenol from *p*-nitrophenyl β-D-glucoside. One unit of enzyme releases 1 μmol equivalent of *p*-nitrophenol per minute. All protein concentrations were determined using the Pierce BCA assay kit (Thermo Fisher Scientific, Waltham, MA, USA) with BSA as the standard.

### Estimation of cellulosomes and cellular protein for monitoring cell growth

Cell growth in BM7CO supplemented with cellulose was monitored based on the increase in the pellet protein concentration. Briefly, cells were lysed in a NaOH/SDS solution of 0.2 N NaOH (Wako Pure Chemical, Osaka, Japan) containing 0.2% (w/v) sodium dodecyl sulfate (SDS; Wako Pure Chemical). Cell debris and residual solids were pelleted from the NaOH/SDS solution and removed by centrifugation (9,700 × *g* for 5 minutes), and the protein concentration in the supernatant was estimated using the Pierce BCA assay kit. Cellulosomes in cell-free broth were isolated from fermenting cultures using the affinity digestion method [44], which is based on binding of cellulosomes to amorphous cellulose. Although over 90% of cellulase activity in the crude supernatant can be recovered by this method, the purified cellulosomal proteins included not only cellulosomes but also non-cellulosomal proteins that can be adsorbed by the amorphous cellulose. Cultures of 1 mL were centrifuged and the cell-free broth was incubated with phosphoric acid-swollen cellulose (PASC; 100 μg/mL of cell-free broth) overnight at 4°C to obtain cellulosomes bound to the cellulose. On the following day, the amorphous cellulose with bound enzymes was centrifuged and washed twice with 50 mM sodium phosphate buffer (pH 7.0). The pellets were resuspended in 1 mL NaOH/SDS. The total protein concentration of the isolated cellulosome samples was determined with the BCA assay kit.

### Biological saccharification using *C. thermocellum* cultures supplemented with β-glucosidases

For subculturing, part of a *C. thermocellum* stock culture was inoculated by syringe into 50 mL of BM7CO medium containing 10 g/L microcrystalline cellulose powder. The subculture was incubated at 60°C for 2 days with rotary shaking at 130 rpm. The subculture was inoculated again by syringe into 50 mL fresh BM7CO medium containing 100 g/L microcrystalline cellulose or 120 g/L (100 g/L glucan equivalent) alkali-pretreated rice straw as the sole carbon sources. After incubation at 60°C for 6 hours with rotary shaking, the cultures were directly supplemented with 30 U/g cellulose or glucan of β-glucosidase (CglT, BglA, or Bgl; 0.03 to 0.05 mg protein/U) in an anaerobic chamber (Hirasawa Co., Ltd. Tokyo, Japan) with a CO_2_-based mixed gas atmosphere. Cultures containing β-glucosidase were incubated again at 60°C for 10 days with rotary shaking at 130 rpm. The concentration of the released sugar was measured by HPLC using culture supernatants obtained by centrifugation (9,700 × *g*, 4°C for 5 minutes). To measure cellulose and glucan concentration in the hydrolyzed residues, aliquots of the separated pellets were dried overnight at 70°C to calculate the percentage of remaining cellulose and the glucan concentration by measuring the weight or following the National Renewable Energy Laboratory (NREL; Golden, CO, USA) chemical analysis procedure described below.

### Compositional analysis of untreated and treated rice straw

The chemical composition of oven-dried natural and alkali-treated rice straw was analyzed following the NREL chemical analysis and testing standard procedure (http://www.nrel.gov/biomass/analytical_procedures.html). Each sample was hydrolyzed with 72% sulfuric acid at 30°C for 60 minutes, followed by 3% sulfuric acid at 121°C for 60 minutes. The autoclaved hydrolyzed solution was neutralized to pH 6.0 with calcium carbonate and vacuum-filtered through a filtering crucible. Mono- and oligosaccharide components were separated on an Aminex HPX-87H column (Bio-Rad Laboratories, Hercules, CA, USA) by HPLC with 1 mM sulfuric acid at a flow rate of 0.6 mL/min on a Prominence instrument (Shimadzu Corp., Kyoto, Japan) operated at 60°C and equipped with a Shimadzu RID-10A refractive index detector. Acid insoluble lignin (Klason lignin) content was defined as the weight of the filter cake (oven-dried at 70°C to constant weight).

### Preparation of alkali-pretreated rice straw

Rice straw (glucan 40.6 ± 0.5%, xylan 17.6 ± 0.1%, arabinan 4.7 ± 0.1%, galactan 3.4 ± 0.1%, and Klason lignin 18.3 ± 0.1%) was purchased from Miyahara (Nagano, Japan). Rice straw was ground through a 0.5 mm mesh screen (ZM-100; Retsch, Haan, Germany) and 30 g of the substrate was added to a 300 mL autoclavable bottle with 150 mL of 10% (w/v) NaOH solution. The bottle was incubated for 5 days at 70°C. The slurry was filtered through a disposable non-woven filter to recover the insoluble solids. The solids were washed with distilled water until the pH of the solid became neutral. The washed solids were dried in an oven at 60°C for 96 hours. After drying, the moisture content of the samples was measured.

### Fermentation tests

The cultures were directly fermented by addition of yeast without any supplements. BM7CO medium adjusted to pH 7.0 with an HCl solution (containing the same concentration of glucose) was inoculated with yeast to obtain the fermentation profile as a reference fermentation test. *S. cerevisiae* strain K3 precultured on YPD medium was washed twice with sterilized water and inoculated into each medium at 5% (v/v) and incubated at 30°C. Samples were analyzed for ethanol using a model GC-2014 gas chromatograph (Shimadzu) with a flame ionization detector (FID).

## Abbreviations

ARS: alkaline-treated rice straw; ATCC: American Type Culture Collection; BSA: bovine serum albumin; BSES: biological simultaneous enzyme production and saccharification; CBM: carbohydrate-binding module; CBM-CglT: Chimera β-glucosidase fused CBM3 from the scaffolding protein CipA into the N-terminal region of CglT; CBP: consolidated bioprocessing; CbP: cellobiose phosphorylase; CdP: cellodextrin phosphorylase; FID: flame ionization detector; HPLC: high-performance liquid chromatography; NPMD: National Institute of Technology and Evaluation Patent Microorganisms Depositary; NREL: National Renewable Energy Laboratory; NRIB: National Research Institute of Brewing; PASC: phosphoric acid-swollen cellulose; PCR: polymerase chain reaction; SD: standard deviation; SDS: sodium dodecyl sulfate; SHF: separate hydrolysis and fermentation; YPD: yeast extract peptone dextrose.

## Competing interests

The authors declare that they have no competing interests.

## Authors’ contributions

PPra carried out the cultivation, collected HPLC data, and helped in the design and interpretation of the experiments. RW helped carry out enzyme assays and participated in the design and interpretation of experiments, and in design and construction of the vectors. CT, PPa, KR, LD, JS, and KS assisted in the design, interpretation, and coordination of the experiments and edited the manuscript. YM helped to draft the manuscript. AK conceived of the project, assisted in the design and interpretation of experiments, and helped to draft and edit the manuscript. All authors read and approved the final manuscript.
